# Biological Roles and Therapeutic Applications of IDH2 Mutations in Human Cancer

**DOI:** 10.3389/fonc.2021.644857

**Published:** 2021-04-26

**Authors:** Jinxiu Guo, Ruyue Zhang, Zhe Yang, Zhenfeng Duan, Detao Yin, Yubing Zhou

**Affiliations:** ^1^ Department of Pharmacy, The First Affiliated Hospital of Zhengzhou University, Zhengzhou, China; ^2^ Henan Key Laboratory for Precision Clinical Pharmacy, The First Affiliated Hospital of Zhengzhou University, Zhengzhou, China; ^3^ Department of Thyroid Surgery, The First Affiliated Hospital of Zhengzhou University, Zhengzhou, China

**Keywords:** IDH2 mutation, cancer metabolism, 2-HG, cancers, IDH2 inhibitors

## Abstract

Isocitrate dehydrogenase (IDH) is a key metabolic enzyme catalyzing the interconversion of isocitrate to α-ketoglutarate (α-KG). Mutations in IDH lead to loss of normal enzymatic activity and gain of neomorphic activity that irreversibly converts α-KG to 2-hydroxyglutarate (2-HG), which can competitively inhibit a-KG-dependent enzymes, subsequently induces cell metabolic reprograming, inhibits cell differentiation, and initiates cell tumorigenesis. Encouragingly, this phenomenon can be reversed by specific small molecule inhibitors of IDH mutation. At present, small molecular inhibitors of IDH1 and IDH2 mutant have been developed, and promising progress has been made in preclinical and clinical development, showing encouraging results in patients with IDH2 mutant cancers. This review will focus on the biological roles of IDH2 mutation in tumorigenesis, and provide a proof-of-principle for the development and application of IDH2 mutant inhibitors for human cancer treatment.

## Introduction

Abnormal metabolism has been established as one of the ten characteristics of cancer cells (1). Metabolic pathways such as glycolysis pathway, tricarboxylic acid cycle and pentose phosphate pathway are important molecular events of life activities. Mutations of key enzyme genes involved in metabolic pathways are the main cause of abnormal metabolism by changing the expression and activity of metabolic enzymes (2). In recent years, genomic mutations of succinate dehydrogenase, pyruvate kinase and isocitrate dehydrogenase have been found in many cancer types (3). Metabolic reprogramming of tumor cells seems to be the result of carcinogenic transformation of human cancer, which may also be one of the causes of oncogenic transformation. In addition, the abnormal accumulation of some metabolites (tumor metabolites) caused by genetic mutations in metabolic genes further supports the importance of metabolic disorders in cancer occurrence and cancer cell survival (4).

**Figure 1:**
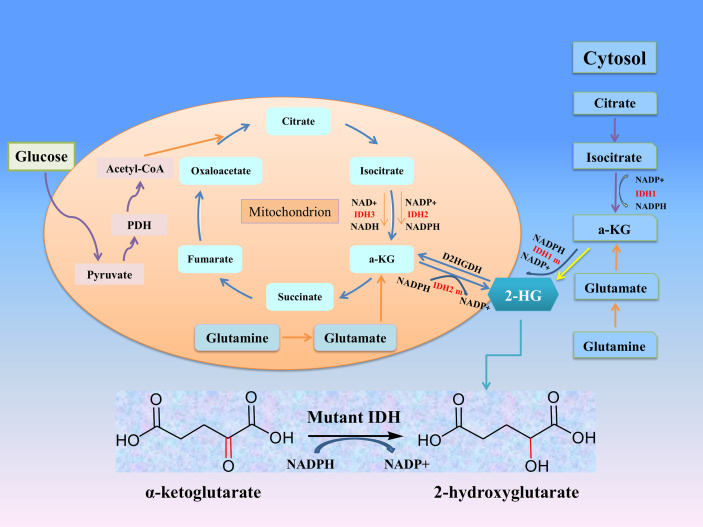
The roles of IDHs in cancer metabolism. Mutation of either IDH1 or IDH2 imparts a neomorphic enzymatic activity upon the encoded enzymes resulting in the ability to convert α-ketoglutarate (α-KG) into the oncometabolite 2-hydroxyglutarate (2-HG), and simultaneously converts NADPH to NADP+.

Isocitrate dehydrogenase (IDH) is an important metabolic enzyme in the tricarboxylic acid cycle, whose mutated genes are associated with a variety of tumors, including acute myeloid leukemia (AML), glioma, cholangiocarcinoma, colon cancer and chondrosarcoma (5). IDH mutation can catalyze the conversion of α-ketoglutarate (α-KG) to 2-hydroxyglutarate (2-HG) (**Figure 1**), and studies have found that the level of 2-HG in cancer patients is higher than that in normal or non-mutated cancer patients (6–8). The increased level of 2-HG and the resulting epigenetic disorders and cell differentiation show the potential carcinogenicity of IDH mutations (9). These mutations attracted considerable interest due to the potential consequences of the neo-enzymatic conversion of α-KG to 2-HG and provided a proof of concept for the development of mutant IDH small molecule inhibitors. The targeted drugs developed according to IDH mutations have entered clinical trials, and some drugs have been involved in clinical treatment (10–12). In this review, we discuss the biological roles and therapeutic applications of IDH2 mutations in human cancers.

## IDH Structure and Functions

IDH metabolic enzyme family includes three subtypes: IDH1, IDH2 and IDH3, which are the most important metabolic enzymes in the Krebs cycle. The structure and function information of IDH metabolic enzymes are introduced below (**Table 1**). The active forms of IDH1 and IDH2, which depend on nicotinamide adenine dinucleotide phosphate (NADP +), are homodimers with similar structures: a large domain, a small domain and a clasp domain (13). And human IDH1 and IDH2 genes are located on chromosomes 2q33.3 and 15q26.1, which encode a 414-amino-acid and a 452-amino-acid (14). IDH3 is a heterooctamer whose activities are dependent on nicotinamide adenine dinucleotides (NAD+), formed by two α subunits (IDH3α), one β subunit (IDH3β), and one γ subunit (IDH3γ), which are encoded by the IDH3A (15q25.1-2), IDH3B (20p13), and IDH3G (Xq28) genes, respectively (15, 16). Subcellular localizedly, IDH1 is mainly located in cytoplasm and peroxidase, while IDH2 and IDH3 are mainly located in mitochondria.

**Table 1:** IDH Structures and Biochemical characteristics.

Subtypes	Isomers	Genes	Chromosome location	Amino acids	Subcellular localization	Co-enzymes	Amino acid substitutions
**IDH1**	Homodimer	IDH1	2q33.3	414 aa	Cytoplasm Peroxidase	NADP+	R132H, R132C, R132L, R132S, R132G
**IDH2**	Homodimer	IDH2	15q26.1	452 aa	Mitochondrion	NADP+	R140Q, R140W R172S, R172T, R172K, R172G, R172I, R172M
**IDH3**	Heterotetramer	IDH3A IDH3B IDH3G	15q25.1-2 20p13 Xq28	366 aa 385 aa 393 aa	Mitochondrion	NAD+	unknown unknown unknown

IDH subtypes can catalyze the oxidation of decarboxylated isocitrate to tricarboxylic acid cycle intermediates α-KG and NADPH, which are involved in other metabolic processes (**Figure 1**). α-KG is an intermediate of the tricarboxylic acid cycle, participates in epigenetic modification as an important cofactor, and regulates hypoxia-inducible factor-1 α and dioxygenase (17). NADPH plays an important role in keeping reduced glutathione and peroxidase, which can maintain the redox balance and protect cells from oxidative damage caused by various cellular stresses. Studies have confirmed that NADPH produced by IDH1 is involved in lipid metabolism and helps cells defend against lipid oxidation-induced reactive oxygen species (ROS) (18). IDH3α was also considered to be an upstream activator of hypoxia-inducible factor-1, which promotes metabolic reprogramming of cancer cells and angiogenesis of malignant tumors by improving the stability and transactivation of hypoxia-inducible factor-1 (19).

## IDH Mutation and Neomorphic Activity

IDH has found frequent genetic mutations in many tumors. IDH1 mutations were first discovered in glioblastoma genome-wide analysis (20). With the development of sequencing technology, IDH1 or IDH2 mutations have been found in various malignant tumors, such as AML, glioma, chondrosarcoma. At present, the IDH3 mutant gene has not been found in tumors, but its abnormal expression was related to the occurrence and development of a few cancers (19). This phenomenon may be related to the unique heterotetramer structure of IDH3 (15). Cancer-related IDH1 and IDH2 mutations occur almost entirely on different arginine residues at the active site of the enzyme. Missense mutation in IDH1Arg132 codon leads to single amino acid substitution. The most common is histidine, but also lead to cysteine, serine, glycine, leucine or isoleucine substitutions (9). The mutation site of IDH1 gene is located at R132, and the mutation sites mainly include R132H, R132C, R 132L, R132S, and R132G (**Table 1**). For IDH2, two mutation hotspots have been described. One was codon 140 (IDH2 R140) and the other at codon 172 (IDH2 R172), including R140Q, R172S, R172T, R172K and R172M (21) (**Table 1**).

**Figure 2:**
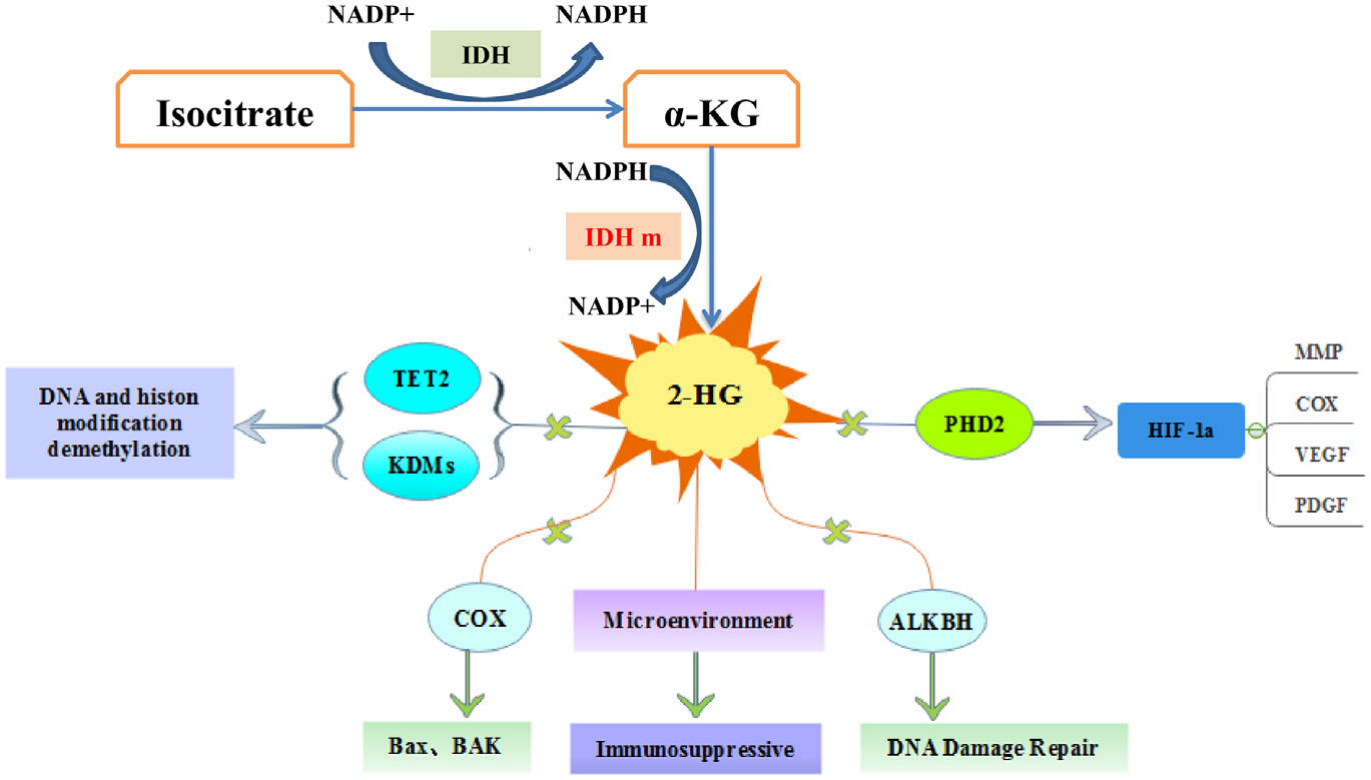
The molecular pathways of IDH mutations in tumorigenesis. 2-hydroxyglutarate competitively inhibits multiple α-ketoglutarate dependent dioxygenases, thereby causing widespread epigenetic changes that result in a global dysregulation of gene expression. 2-HG inhibits prolyl hydroxylases (PHD) and increases HIF-1α stabilization and result in impairment of collagen maturation. 2-HG can promote apoptosis by inhibiting cytochrome c oxidase (COX), and associate with tumor microenvironment.

After IDH mutation, the catalytic activity decreased, and the yield of corresponding enzymatic reactants NADPH and α-KG decreased, but at the same time, a new enzymatic activity was obtained, which catalyzed NADPH and a-KG to produce a new enzyme active metabolite 2-HG (22) (**Figure 1**). The expression level of 2-HG is related to different mutation sites, and IDH2 R172 mutations usually lead to a very high level of 2-HG accumulation (23, 24). At present, 2-HG is considered to be a tumor metabolite because it is involved in a variety of biological processes related to tumorigenesis (25, 26) (**Figure 2**). Although more than 60 different α-KG-dependent dioxygenases have been described, there seem to be two main targets for 2-HG: the KDM family of histone lysine demethylases and the TET family of 5-methylcytosine hydroxylase (27). Because of its structural affinity to α-KG, 2-HG competitively inhibits histone demethylase and Tet family methylcytosine hydroxylase (25), resulting enzyme block leads to increased histone H3 lysine methylation and global DNA hypermethylation (28, 29). Hypermethylation has been shown to lead to changes in gene expression, many of which are related to cell differentiation. The decrease of α-ketoglutarate also leads to the decrease of proline hydroxylase and the up-regulation of HIF-1 α, which destroys the adaptability to hypoxia (30, 31). In addition, 2-HG can increase the level of vascular endothelial growth factor (VEGF) secreted by cancer cells and promote endothelial cell proliferation in a concentration-dependent manner. Some results suggest that 2-HG induces angiogenesis activity and increases MMP2 activity through VEGFR2 signal (32). The researchers found that 2-HG not only directly regulates a variety of α-KG-dependent dioxygenases, but also directly inhibits cytochrome c oxidase (COX) in the mitochondrial electron transport chain (ETC). This leads to the activation of pro-apoptotic Bax and BAK, which triggers hypoxia-induced cell death (33). 2-HG released from the microenvironment may also alter the function of non-tumor cells around the tumor, such as neurons and immune cells (34). In fact, there is now direct evidence that 2-HG accumulation plays an immunosuppressive role. In the presence of high levels of 2-HG, the proliferation ability of activated CD4+ and CD8+T cells decreased (35).

## IDH2 Mutations in Human Cancer

**Figure 3:**
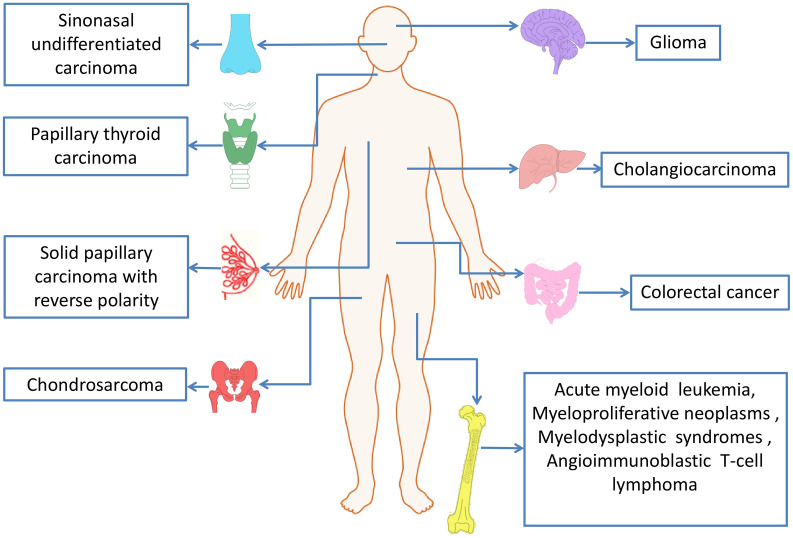
IDH2 mutations in human cancers.

IDH mutations have been detected in multiple tumor types, including various solid tumors and several myeloid malignancies. High frequent mutations in IDH2 have been found in AML, glioma, chondrosarcoma, angioimmunoblastic T cell lymphoma (AITL) and solid papillary carcinoma with reverse polarity (SPCRP), and IDH2 mutations were also reported in other malignant tumors (**Table 2**, **Figure 3**).

**Table 2:** IDH2 Mutations in Human Cancer.

Cancer types	Mutation modes	Co-occurring mutations	Detection methods	Refs
**AML**	R140L R140G R140Q R140W R172K R172S R172W	NPM1 DNMT3A SRSF2 FLT3-ITD ASXL1 NRAS RUNX1	Next-generation sequencing Gene scan Quantitative PCR Sanger sequencing Direct sequencing	(36)
**Glioma**	R172G R172M R172S R172K R172W	TP53 PTEN EGFR CDKN2A CDKN2B	Genomewide mutation analysis The direct DNA sequencing Mutation-specific mAbs	(37, 38)
**DDCHS**	R172S R172T R172G R172M	TERT TP53 CDKN2A/2B	Sanger sequencing qPCR genotyping MSK-IMPACT sequencing	(39–41)
**AITL**	R140G R172K R172G R172S R172T	TET2 DNMT3A	Sanger sequencing Sequenom	(42)
**SPCRP**	R172S R172T R172G R172I R172W	TET2 PIK3CA	Next-generation sequencing Sanger sequencing SNaPshot genotyping Whole exome and targeted massively parallel sequencing	(43, 44)
**SNUC**	R172M R172S R172T R172G	TP53 PIK3CA	Next-generation sequencing Sanger sequencing MSK-IMPACT sequencing	(45)

### AML

Whole-genome sequencing of IDH2 gene mutations have found that the genes encoding epigenetic regulators in AML samples are frequently mutated, about 20% of patients with AML have IDH gene mutations (36, 46). IDH1 and IDH2 mutations occur at comparable frequencies in AML, but no patient had both IDH1 and IDH2 mutations (37). IDH2 mutation has the clinical characteristics of older age, lower white blood cell count; higher platelets and NPM1 mutations in patients with AML (47, 48). In addition, researchers found that R172 IDH2 mutations had potential adverse prognostic significance, and that R172 IDH2 mutations are mutually exclusive with any other prognosis-related mutations. IDH2 mutant AML patients are associated with a resistance to treatment as illustrated by a low rate of CR and a high RR (36, 47). A predictive analysis of the leave-one-out cross-validation further explains this phenomenon (47). The results showed that the genes up-regulated in patients with R172 IDH2 mutation were ID1, ABCB1 and KRAS2 associated with the poor prognosis of AML. The down-regulated genes are KYNU involved in NAD cofactor biosynthesis, SUCLG2 involved in the Krebs cycle; and CD93 involved in the regulation of phagocytosis and angiogenesis of apoptotic cells. These genomic changes in AML usually have a clear correlation with the clinical diagnosis and prognosis of patients, and are widely used to guide the clinical use of drugs and indicate the prognosis of AML.

With the continuous deepening of understanding of the role of IDH2 mutations and their metabolites in AML, IDH2 inhibitor targeted therapy for AML patients has gradually been applied in clinical practice. Inhibitors of mutant IDH2 may reduce the level of 2-HG to reverse cell differentiation (49), and indirectly destroy the bone marrow microenvironment induced by 2-HG by blocking the proliferation of AML cells (21). Enasidenib (AG-221) is the first IDH inhibitor approved by the FDA for the treatment of relapsed or refractory acute myeloid leukemia (RR-AML) with IDH2 mutations and achieved good therapeutic effects (50).

### Glioma

Somatic mutations of IDH2 were first discovered in gliomas (51). Gliomas are a large and diverse group of primary brain tumors that include those that are diffusely infiltrative and others that are well-circumscribed and low grade (52). More recently, based on the sequencing results of large samples, it was found that about 60% to 80% of patients with grade II and III gliomas and most patients with secondary glioblastoma (GBM) had somatic mutations encoding isocitrate dehydrogenase genes, mainly IDH1 R132 mutation (53, 54). IDH2 mutations are mutually exclusive with IDH1 mutations, and thus far, have been found rarely (3.3%) in WHO grade II or III gliomas (55) and much rarely (less than 1%) in GBMs (38). In contrast, IDH2 mutations are most common in AML and residue R140, while IDH2 R140 mutations have not been detected in early gliomas or cartilage tumors

The prognostic effect of tumor was related to many factors. The prognosis of gliomas with IDH mutation was better than that of AML with IDH mutation, which is related to the difference of susceptible mutation sites between the two kinds of tumors. In gliomas, there is a close relationship between grade and prognosis. The latest study found that the prognosis of grade II and grade III was good, while the prognosis of grade IV was controversial (40). At the same time, compared with wild-type IDH tumors, glioma patients with IDH1/2 mutation had better prognosis and better therapeutic effect as they were younger. Neuropathologic assessment of gliomas increasingly relies on ancillary testing of molecular alterations for proper classification and patient management. Lower-grade gliomas with both an IDH mutation (a mutation in either IDH1 or IDH2) and deletion of chromosome arms 1p and 19q (1p/19q codeletion), which occurs most often in oligodendrogliomas, had better responses to radio chemotherapy and were associated with longer survival than diffuse gliomas without these alterations (56). The presence of IDH mutations failed to demonstrate a significant influence on survival in the multivariate analysis of low-grade astrocytomas (LGA) patients. Early RT appears to be beneficial only LGA patients with IDH-mutations (53). Lower-grade gliomas with an IDH mutation either had 1p/19q codeletion or carried TP53 mutation (56). Among patients with IDH mutant gliomas, those in the double-mutant subset had better survival and a lower incidence of malignant degeneration than those in the IDH-only subset (57). Noteworthily, although IDH mutation is associated with longer patient overall survival, IDH mutant companied with MGMT methylation subsets consistently showed higher risks of malignant transformation in low-grade glioma, compared to IDH wild type (58).

### Chondrosarcoma

Recently, researchers have discovered IDH2 R140 mutation in three advanced chondrosarcoma samples (59). Patients with chondrosarcoma carry more than 50% of IDH1/2 mutant heterozygotes (41), and IDH1 R132 is the most common mutation, followed by IDH2 R172. Interestingly, in chondrosarcomas, the frequency of IDH2 mutations increases with grade. IDH2 is present in 22% of high-grade chondrosarcomas and only about 7% in low-grade tumors (59). Therefore, it is worth noting that many studies have suggested that the grade of chondrosarcoma is related to many tumor-related factors. IDH1/2 mutant cells need α-KG to produce 2-HG, which can be produced by glycolysis and glutamine decomposition (60). A comparative study found that compared with low-grade chondrosarcoma, the expression of glycolysis-related genes is increased, and glutaminase is also high in high-chondrosarcoma, but glutamine degradation has nothing to do with IDH1/2 mutation (61). Hypermethylation of the nicotinic acid phosphoribosyl transferase (NAPRT) promoter was observed in advanced chondrosarcom. TP53 mutates in approximately 30% of chondrosarcomas (62), and this mutation mainly occurs in advanced chondrosarcomas. Some genes encoding energy metabolic components will change with increasing grade, but due to the influence of a series of complex factors, such as tumor type, tumor microenvironment and so on, whether the difference of high-grade chondrosarcoma is related to IDH mutation needs further study.

Dedifferentiated chondrosarcoma (DDCHS) is a kind of high-grade chondrosarcoma, which is transformed from low grade and has strong invasiveness (63). The frequency of IDH2 mutations in dedifferentiated chondrosarcoma is much higher than that of other chondrosarcomas (64). Researchers have found that up to 76% of IDH heterozygous mutations in DDCH, 39.1% of which are IDH2 mutations, 2-HG levels in tumor tissues are significantly higher than normal tissues (64). Given the high incidence of IDH2 mutations in DDCHS, IDH2 can be used as a diagnostic marker for DDCHS. And the distinction between DDCHS and osteosarcoma is very important for clinical treatment, IDH2 mutation analysis can be regarded as a suitable auxiliary test for the diagnosis of DDCHS (63).

Although IDH1 and IDH2 mutations are associated with better OS in glioma patients, it is controversial whether OS has a good correlation with chondrosarcoma patients (65, 66). A recent study showed that the OS of chondrosarcoma patients with IDH2 mutation was significantly shorter than that of patients without mutation. IDH mutation status is not related to overall survival, but IDH2 mutation is associated with longer recurrence-free survival (RFS) and metastasis-free survival (MFS) in high-grade chondrosarcomas (41). The coexistence of TERT mutations, CDKN2A/2B and TP53 changes in high-grade chondrosarcomas may explain this phenomenon (41).

### Angioimmunoblastic T-Cell Lymphoma (AITL)

AITL is a subtype of PTCL, which is characterized by high frequency of epigenetic factor overlap mutation and poor prognosis (67). TET2, RHOA, IDH2 R172 and DNMT3A are widespread genetic damages in AITL, in which the mutation rate of TET2 is the highest, and IDH2 R172 mutation can be different from other entities of PTCL (42). At present, there are only IDH2 R172 heterozygous mutations in AITL, and there are no other IDH mutants. Recently, IDH2 R172 mutations are described in AITL, and the prevalence rate is about 20%-45% (68). Sanger sequencing was used to detect mutations in all exons, and the mutation spectrum showed that IDH2 mutations at R172 were R172K, R172S, R172T and R172G (69). In patients with IDH2 mutated angioimmunoblastic T-cell lymphoma, the levels of intracellular and plasma 2-HG were not the same thing, and the level of intracellular 2-HG was higher than that in plasma (70). Some studies have provided another evidence for this: the R172 mutation in IDH2 has a greater ability to produce 2-HG in lymphoid cells and can damage the development of lymphoid cells, which may explain the advantage of this mutation in AITL (71). In addition, RHOAG17V and TET2 mutations coexist frequently in AITL patients with IDH2 mutations, suggesting that multiple mutations may work together to drive the transformation of this cell type (43). The overlapping mutations of epigenetic factors may be closely related to the poor prognosis of AITL. Further study of the combined effect of TET2, IDH2 and RHOA mutations may be more beneficial to the treatment of AITL patients.

### Solid Papillary Carcinoma With Reverse Polarity (SPCRP)

SPCRP, also known as resembling the tall cell variant of papillary thyroid neoplasms (BPTC),is a rare breast cancer subtype with unusual histopathological features (44). SPCRPs are uniquely characterized by harbor recurrent IDH2 R172 hotspot mutations or TET2 mutations, and IDH2 R172 hotspot mutations often in combination with mutations in PI3K pathway genes, in particular in the form of PIK3CA hotspot mutations. Studies have shown that IDH2 R172 hotspot mutations coexisted with PIK3CA mutations in 50% of cases (72). IDH2 R172 mutations in solid papillary carcinoma with reverse polarity most of which are in the form of R172S or R172T mutations, IDH2 R172G, R172I and R172W mutations have also been identified.

Through immunohistochemical characterization, Complete exon group (WES), targeting and Sanger sequencing of 13 SPCRPs, 10 SPCRPs were found to have hot spot mutations at R172 of IDH2, of which 8 SPCRPs showed pathogenic mutations affecting PIK3CA or PIK3R1, and one IDH2 wild type SPCRP contained TET2Q548 truncated mutation and PIK3CA H1047R hot spot mutation. At the same time, functional studies also showed that a high concentration of 2-HG was detected in the SPCRP with IDH2 mutation, and global DNA hypermethylation and H3K27 trimethylation were observed in the SPCRP with IDH1/IDH2 mutations (72), which was consistent with the cancer characteristics of IDH1/IDH2 mutation. There was research reported that researchers sequenced the whole exon of 9 cases of SPCRP and used IDH1/IDH2 mutant (R132/R172) antibody in resected specimens for immunohistochemical analysis (73). At present, the main detection methods of IDH mutation gene are immunohistochemical detection and gene sequencing. SPCRP with IDH2 R172 hot spot mutation can be detected with high sensitivity and specificity by immunohistochemical staining with monoclonal antibody against IDH2 R172 mutation (74, 75). In view of the development of IDH2 mutation inhibitors, IDH2 mutations in SPCRP may become a new target for breast cancer treatment and intervention.

### Other Cancers

In addition to the above tumors, IDH2 mutations in sinonasal undifferentiated carcinoma (SNUC) are also common, with a mutation rate of about 48-82.4% (45, 75). The currently reported mutation is the R172 mutation, and no R140 mutation has been found. TP53 and PI3K mutations in cancer patients with IDH2 mutations are a common phenomenon. Although IDH2-mutated SNUC were associated with a trend of improved free survival and overall survival, such trend did not reach significant level (75).

IDH2 mutations have been reported in cholangiocarcinoma, an aggressive cancer associated with epithelial cells lining the bile duct. IDH2-R172K mutations in adult hepatocytes can cause the production of 2-HG and create a transformable pre-tumor state through other carcinogenic changes. The expression of IDH2 R172K is negatively correlated with OS, which is specifically expressed as a precursor oval cell population expansion and multistage intrahepatic cholangiocarcinoma (IHCC) (39). In addition, in patients with IDH1/2 mutant IHCC, the circulating level of 2-HG is directly related to tumor burden (76). IDH2 mutations in lower frequencies have also been identified in other malignant tumors, including myeloproliferative neoplasms (MPN) and myelodysplastic syndromes (MDS) (77).

Although IDH2 mutation sites were not found in some cancers, the levels of some landmark metabolites changed significantly after IDH2 overexpression. Some results demonstrate the potential role of IDH2 in the biological mechanisms and progression and also indicate IDH2 as oncogene. Clinical specimens and cell experiments of papillary thyroid carcinoma provide effective evidence for this (78). IDH2 is associated with the occurrence and prognosis of gastric cancer (79). It was found that the expression level of IDH2 in gastric cancer was significantly decreased, and the low expression level of IDH2 was significantly correlated with the survival rate of patients with gastric cancer. The overexpression of IDH2 can increase the level of 5hMC in gastric cancer cells, while the low expression of IDH2 may lead to the depletion of 5hMC in gastric cancer cells. The down-regulation of IDH2 inhibited the growth and movement of gastric cancer cells. This phenomenon suggests that IDH2 plays a carcinogenic role in gastric cancer cells, which is similar to the effect of IDH2 on low-grade gliomas.

## IDH2 Mutant Inhibitors

In view of the important roles of mutant IDH1, IDH2 and their products in tumorigenesis and progression, another strategy of tumor therapy is to target mutant enzymes and products. In the past few years, a variety of IDH mutant enzyme inhibitors have been developed, including one PAN inhibitor and several inhibitors targeting specific IDH mutant subtypes (**Table 3**). A large number of preclinical studies have shown that IDH mutant inhibitiors can significantly reduce the level of 2-HG and have great effects on cell metabolism, growth and tumorigenicity. For many of these inhibitiors, the eutectic structure of enzyme-binding inhibitors has been determined by X-ray crystallography, and their inhibition mechanism has been elucidated at the molecular level. Most inhibitors regulate the activity of the enzyme through allostery, rather than competing for the binding of the substrate and the active site. Several of them have been used in clinical trials, and Enasidenib (AG-221) and Ivosidenib (AG-120) have been approved by FDA for human cancer treatment (**Table 3**).

**Table 3:** Development of IDH Mutant Inhibitors for Cancer Treatment.

Inhibitors	Targets	Cancers with IDH mutation	R & D status	Clinical trial identifiers
**AG-221** **(FDA approved)**	IDH2 mutant	Glioma Angioimmunoblastic T-cell lymphoma Cholangiocarcinoma Chondrosarcoma MDS AML	Phase 1/2 Phase 1/2 Phase 1/2 Phase 1/2 Phase 3 Phase 3	NCT02273739 NCT02273739 NCT02273739 NCT02273739 NCT03839771 NCT03839771
**AG-120** **(FDA approved)**	IDH1 mutant	AML Glioma Chondrosarcoma MDS Chondrosarcoma Cholangiocarcinoma	Phase 3 Phase 2 Phase 2 Phase 2 Phase 2 Phase 3	NCT03839771 NCT04056910 NCT04278781 NCT04044209 NCT04278781 NCT02989857
**AG-881**	IDH1/IDH2 mutant	AML Glioma	Phase 1 Phase 3	NCT02492737 NCT04164901
**IDH305**	IDH1 mutant	AML Glioma MDS	Phase 1 Phase 2 Phase 1	NCT02826642 NCT02977689 NCT02381886
**BAY1436032**	IDH1 mutant	Solid Tumors AML	Phase 1 Phase 1	NCT02746081 NCT03127735
**FT-2102**	IDH1 mutant	AML MDS Glioma Chondrosarcoma	Phase 1/2 Phase 1/2 Phase 1/2 Phase 1/2	NCT04013880 NCT04013880 NCT03684811 NCT03684811
**AGI-5198**	IDH1 mutant	Glioma	Preclinical	/
**AGI-6780**	IDH2 mutant	AML	Preclinical	/
**MRK-A**	IDH1 mutant	Glioma	Preclinical	/
**GSK321**	IDH1 mutant	AML	Preclinical	/
**GSK864**	IDH1 mutant	AML Glioma	Preclinical	/

### AG-221

**Figure 4:**
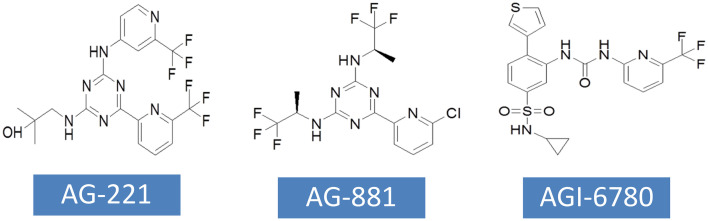
The chemical structures of currently developed inhibitors targeting IDH2 mutations.

AG-221 has been approved by FDA for the treatment of RR-AML with IDH2 mutations. AG-221 was obtained through high-throughput screening and chemical structure optimization (80). Its precursor form is a triazine compound (**Figure 4**). After a series of chemical modification steps, the oral bioavailability, solubility and clearance rate of the precursor were optimized, and AG-221 which can be used in clinical development was obtained (81). Initial preclinical studies have shown that AG-221 can reduce the secretion of 2-HG in IDH2 R140Q mutant cells, thereby inhibiting cell proliferation, inducing cell differentiation (82), reversing histone hypermethylation associated with IDH2 mutations. In vivo experiments, oral administration of AG-221 can significantly improve the survival rate of nude mice. Based on the good preclinical results of AG-221, the clinical trial of AG-221 started quickly. In clinical trials, AG-221 has been used in patients with advanced hematological malignant tumors and known IDH2 mutations, and clinical trials have been conducted to test the safety and effectiveness of AG-221 (50, 83). The plasma concentration of AG-221 was stable, and the content of 2-HG decreased most significantly when the dose was 100mg·d-1. The median survival time of refractory recurrent acute myeloid leukemia was 9.3 months, and the median survival time of patients with complete remission and partial remission after AG-221 was 19.7 months and 14.4 months, respectively. The study by Amatangelo et al. have shown that myeloblast differentiation, neutrophil recovery and platelet recovery are obvious in AML patients treated with IDH mutation inhibitors (83). The reduction of 2-HG level eliminates the obstacle of myeloid differentiation and promotes the differentiation of primordial cells expressing mutations (84).

It has been recently reported that in a small number of patients with recurrent or refractory AML, treatment with mutant IDH1 or mutant IDH2 inhibitors may lead to clinical differentiation syndrome, which is characterized by leukocytosis and exuberant neutrophil recovery. In addition, the most common treatment-related adverse events were indirect hyperbilirubinemia and nausea, and differentiation syndrome associated with IDH inhibitors was one of the most common highly adverse events (85). These findings suggest that clinicians must be aware that mutated IDH inhibitors may cause complications such as differentiation syndrome in AML patients. Since Enasidenib received approval for AML indications for recurrent/refractory IDH2 mutations in 2017, there have been many clinical trials investigating the efficacy of mutant IDH2 inhibitors (mainly Enasidenib) in different AML subtypes and solid tumors (**Table 3**). The combination of AG-221 and other inhibitors has become one of the main research directions.

### AG-881

AG-881 is the first PAN inhibitor developed by Celgene in cooperation with Agios Pharmaceuticals (86) (**Figure 4**). AG-881 is a small molecular inhibitor of IDH1 and IDH2 mutations, oral administration can reduce the formation of tumor metabolite 2-HG. At present, there are two mechanisms of AG-881 inhibition. One inhibition mechanism is that the triazine part of AG-881 can effectively inhibit the allosteric activity of the enzyme, and crystallographic studies have shown that AG-881 binds to the allosteric pocket of the two enzymes and locks the enzyme in an inactive conformation (86). Another inhibition mechanism is the direct interaction between mutant IDH1 inhibitors and Asp279, and the binding of compounds prevents the catalytic active conformation of Mg/Mn2+ binding with Asp279 from playing a role. Interestingly, the effects of AG-881 on these two mutations are different. Existing studies have confirmed that the binding of AG-881 to IDH1 mutant is more effective than the interaction between AG-881 and IDH2 mutant, especially with IDH1 R132H (87). In preclinical studies, primary human AML cells treated with AG-881 *in vitro* can induce myeloid differentiation, and it also shows good blood-brain barrier penetration in rodents. Currently, AG-881 is conducting a phase I clinical study in patients with advanced solid tumors and advanced hematological malignant tumors in muIDH1/2.

### AGI-6780

AGI-6780 is a urea sulfonamide inhibitor obtained by high-throughput screening and drug design (88). It has been found that AGI-6780 is an allosteric inhibitor, which binds to the substrate noncompetitively and plays a role at the dimer interface, which can effectively and selectively inhibit IDH2 R140Q mutation (88). At present, the research on the biological effects of AGI-6780 is limited to the environment related to acute myeloid leukemia (89). There are two confirmed mechanisms for AGI-6780: one is to induce the differentiation of TF-1 erythroleukemia cells and primary human AML cells, and the other is to reverse the hypermethylation of DNA and histones induced by 2-HG (49). Although AGI-6780 was developed early, its functions are limited.

Additionally, the potential drug side effects and treatment resistance has promoted the production of novel IDH2 mutant inhibitors with specific targeting and improved selectivity. Gao et al. reported TQ05310 as mutant IDH2 inhibitor targeting both IDH2 R140Q and IDH2 R172K mutants, and found that TQ05310 inhibited mutant IDH2 enzymatic activity, suppressed 2-HG production and induced differentiation in cells expressing IDH2 R140Q and IDH2 R172K, but not in cells expressing wild-type IDH1/2 or mutant IDH1. TQ05310 also had favorable pharmacokinetic characteristics in a tumor xenografts model (90). A heterocyclic urea amide compound, CP-17, was identified as a potent inhibitor of IDH2 R140Q mutant by in silico screening and enzymatic assay, exhibited excellent inhibitory activity against IDH2 R140Q and showed dramatic improvement over previously developed inhibitors such as AGI-6780 and AG-221. Cellular assay results demonstrated that CP-17 inhibited intracellular 2-HG production and suppressed the proliferation of TF-1 erythroleukemia cells carrying IDH2 R140Q mutant (91). Li and colleagues designed and synthesized a series of novel 2-arylbenzimidazoles and evaluated their inhibitory activity against IDH2 R140Q mutant. The preliminary results indicated that four compounds 7b, 7c, 7m and 7r displayed the potent inhibitory activity against IDH2 R140Q mutant. Among them, compound 7c showed the highest inhibitory activity, which was more active than positive control AG-221, according to the IC_50_ values (92). These selective IDH2 mutant inhibitors will provide promising candidates for the clinical development of IDH2-targeted drugs.

## Conclusion

A number of studies have provided strong evidence for the carcinogenic potential of IDH2 mutations, leading to the production of tumor metabolite 2-HG, which changes epigenetic regulation, cancer cell differentiation and cell metabolism. In addition, the mutation status of IDH2 genes is associated with the prognosis of tumor patients. Preclinical studies *in vitro* and *in vivo* have shown that inhibition of mutated IDH2 enzyme can reduce the level of intracellular 2-HG, reverse the loss of epigenetic control, and release the differentiation block of cancer cells. More importantly, the selective IDH2 mutant inhibitor AG-221 has achieved promising results in clinical practice. Therefore, further study on the biological roles of IDH2 mutations in tumorigenesis and development of potent IDH2 mutant inhibitors will improve the clinical treatment of certain cancer types.

## Author Contributions

This review was designed by YZ and JG. The original manuscript was written by JG. The data was analyzed by RZ and ZY. ZD, DY and YZ are responsible for supervising and revising the manuscript. Funding was obtained by YZ and DY. All authors contributed to the article and approved the submitted version.

## Funding

This work was supported by the National Natural Science Foundation of China (No.: 81402266), Henan Scientific and Technological Research Projects (No.: 202102310044), the Outstanding Young Talent project of Scientific and Technological Innovation in Henan Health (No.: YXKC2020032), and the Medical Science and Technology Research Projects of Henan Province (No.: SBGJ202002082). Support has also been provided by the Clinical Pharmacy Branch of Chinese Medical Association-Wu Jieping Medical Foundation (No.: 320.6750.19090-3), Bethune exploration Project in Pharmaceutical Research (B-19-H-20200622), the International Talent Cooperation Project of Henan Province (No.: GH2019015), Major Scientific Research Projects of Traditional Chinese Medicine in Henan Province (No.20-21ZYZD14), and Cultivation of Young and Middle-aged Health Science and Technology Innovation Leading Talents in Henan Province (YXKC2020015).

## Conflict of Interest

The authors declare that the research was conducted in the absence of any commercial or financial relationships that could be construed as a potential conflict of interest.
